# Quantum loss sensing with two-mode squeezed vacuum state under noisy and lossy environment

**DOI:** 10.1038/s41598-023-32770-7

**Published:** 2023-04-12

**Authors:** Sang-il Park, Changsuk Noh, Changhyoup Lee

**Affiliations:** 1 Kyungpook National University, Daegu, 41566 Korea; 2 Korea Research Institute of Standards and Science, Daejeon, 34113 Korea

**Keywords:** Quantum physics, Quantum metrology

## Abstract

We investigate quantum advantages in loss sensing when the two-mode squeezed vacuum state is used as a probe. Following an experimental demonstration in PRX 4, 011049, we consider a quantum scheme in which the signal mode is passed through the target and a thermal noise is introduced to the idler mode before they are measured. We consider two detection strategies of practical relevance: coincidence-counting and intensity-difference measurement, which are widely used in quantum sensing and imaging experiments. By computing the signal-to-noise ratio, we verify that quantum advantages persist even under strong thermal background noise, in comparison with the classical scheme which uses a single-mode coherent state that directly suffers from the thermal noise. Such robustness comes from the fact that the signal mode suffers from the thermal noise in the classical scheme, while in the quantum scheme, the idler mode does. For a fairer comparison, we further investigate a different setup in which the thermal noise is introduced to the signal mode in the quantum schemes. In this new setup, we show that the quantum advantages are significantly reduced. Remarkably, however, under an optimum measurement scheme associated with the quantum Fisher information, we show that the two-mode squeezed vacuum state does exhibit a quantum advantage over the entire range of the environmental noise and loss. We expect this work to serve as a guide for experimental demonstrations of quantum advantages in loss parameter sensing, which is subject to lossy and noisy environment.

## Introduction

Optical spectroscopy is a powerful experimental technique for investigating various physical systems and is applied to diverse areas of science and technology from both fundamental and practical viewpoints^1–6^. In transmission spectroscopy, for example, one shines a light beam on an analyte and measures the intensity of the transmitted light. The measurement followed by a post-processing step yields the transmittance *T* of an analyte with a noise $\Delta T$ representing the uncertainty in the estimated value of *T*. In most cases, the figure of merit used to quantify the quality of the measured signal is the signal-to-noise ratio (SNR) and thus it is of utmost importance to increase the SNR. To this end, one can simply crank up the power of the illuminating light, thereby increasing the intensity of the transmitted light. This is not always acceptable, however, due to optical damages that could occur when a photo-sensitive analyte is under inspection^7^. In such cases, the energy of the illuminating beam is constrained and the SNR cannot be increased arbitrarily. Here, quantum sensing techniques developed over the past few decades can be used to further enhance the SNR. By carefully choosing the quantum state of the probe beam as well as the measurement performed on the scattered light, one can enhance the SNR without increasing the intensity of the probe beam^8^. Among various types of parameter estimation problems treated in the framework of quantum sensing, the so-called loss sensing, understood to be conjugate to phase sensing, is directly related to optical spectroscopy in that it aims to precisely measure the amount of energy that is lost during propagation through an analyte^9–15^.

One of the most practical states used in quantum sensing is the two-mode squeezed vacuum (TMSV) state, which can be generated via spontaneous parametric down-conversion in a nonlinear crystal^16,17^. The TMSV state possesses strong quantum correlations between the two modes in photon number, frequency, time, and position. By exploiting such correlations, the TMSV state has proven to be extremely useful in many quantum technological applications^1,18–23^ including single^9–14^ and multi-parameter loss sensing^15^. In particular, the recent experimental work demonstrated that a loss sensing scheme that uses the TMSV state along with coincidence detection is more robust to thermal noise compared with a classical scheme that uses the coherent state^1^. In other words, the SNR for the quantum scheme is larger than that of the classical scheme in the presence of large thermal noise. Such behavior is very interesting because classical schemes usually outperform quantum schemes when noise dominates.

In the first part of this work, we perform a quantum theoretical analysis of the experimental setup studied in Ref. ^1^, by comparing the SNRs for the quantum and classical schemes. In the quantum scheme, the TMSV state is used along with a coincidence-counting measurement, while in the classical scheme the coherent state is used along with the intensity measurement, i.e., photon counting. Our calculations show that a significant advantage is observed when there is large thermal noise, supporting the conclusion of the experimental work. We also investigate the performance of an alternative quantum scheme where the number-difference measurement is employed and show that it is the preferred scheme when thermal noise is weak and the sample transmission is large. We then compare the aforementioned particular detection schemes against the optimal ones that can be determined using quantum estimation theory, both for the classical and quantum setups.

In the second part, noting that the quantum scheme’s robustness against noise stems from the difference in the way the noise is introduced in the classical and quantum setups, we move on to investigate a new quantum setup, in which the noise is introduced in a fairer fashion. Analyzing the performance of the TMSV state under the new setup, we find that the classical scheme is preferred over a much larger parameter regime in the large noise limit, in contrast to the original setup. Even in the parameter regime in which a quantum scheme outperforms the classical scheme, the advantages are significantly diminished.

## Background

**Figure 1:**
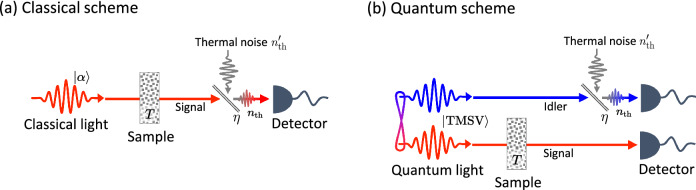
Diagrammatic illustrations of the classical and quantum setups investigated in Ref.^1^. (**a**) In the classical scheme, a coherent state probe first passes through the sample and then experiences a thermal noise with an average photon number $n_\mathrm{th}$. The latter is modeled by a fictitious beam splitter with transmittance $\eta$, into which a thermal noise $n_\text {th}'$ is injected from the background. (**b**) In the quantum scheme, the signal mode of a two-mode squeezed vacuum (TMSV) probe passes through the sample, while the idler mode experiences the thermal background.

The classical and quantum spectroscopy setups of Ref.^1^ are illustrated schematically in Fig. 1. To generate a classical probe, a halogen lamp was used in Ref. ^1^, which generates a multi-mode thermal light. Instead of using a thermal state as a probe, however, we consider a coherent state as the classical probe, which is the most commonly used classical benchmark in quantum sensing and outperforms the thermal states in the sensing tasks described below. This leads us to set the classical scheme, where the probe field is prepared in a coherent state and passes through the sample and a thermal background as shown in Fig. 1a. The resulting output is then detected by a photon counter. In the quantum scheme, the probe field is prepared in the TMSV state $\cosh ^{-1}{r}\sum _{n=0}^\infty e^{in\theta }\tanh ^{n}{r}|n,n\rangle$ (with $\theta =0$ in the rest of this work), and its signal beam goes through the sample while the idler beam experiences the thermal background. The resulting two modes are subsequently detected by separate photon counters.

The sample is modeled by a beam splitter with transmittance *T*, with the vacuum entering the unused port, and the thermal noise is modeled by another beam splitter with transmittance $\eta$ with a thermal field entering the unused port. To ensure that the number of thermal photons entering the detectors is fixed to $n_\mathrm{th}$ independently of $\eta$, the input thermal state entering the unused port is assumed to have the photon number $n_\mathrm{th}' = n_\mathrm{th}/(1-\eta )$. In the experiment, both the input and the thermal lights were multi-mode and the frequency dependence of the sample transmittance was kept into consideration. For simplicity, we use a single mode description in which only a narrow frequency window is considered. There is no loss of generality, however, since by varying the central frequency of the probe beam one recovers the full spectral dependence of the sample transmittance *T*. Assuming that the thermal noise parameters are known beforehand, an unbiased estimator of the transmittance *T*–that yields the estimate *T* from the intensity measurement $a_\text {out}^\dagger a_\text {out}$–in the classical scheme can be set as

1 $$\begin{aligned} \hat{T}_\mathrm{C}=\frac{a_\mathrm{out}^\dagger a_\mathrm{out}-n_\mathrm{th}}{\eta n}, \end{aligned}$$

where $a_\mathrm{out}$ is the annihilation operator of the field entering the photon counter and *n* is the mean photon number of the probe beam (see Supplementary Information). The expectation value and the variance of the estimator are then $\langle \hat{T}_\mathrm{C} \rangle = T$ and

2 $$\begin{aligned} \langle ( \Delta \hat{T}_\mathrm{C})^2 \rangle = \frac{T(2n_\mathrm{th}+1) + \frac{n_\mathrm{th}(n_\mathrm{th}+1)}{\eta n}}{\eta n}, \end{aligned}$$

respectively.

In the quantum setup, on the other hand, we consider two different measurement schemes. The first scheme is the coincidence-counting scheme used in Ref. ^1^, whose measurement operator can be written as $a_\mathrm{out}^\dagger a_\mathrm{out} b_\mathrm{out}^\dagger b_\mathrm{out}$. The second scheme is the number-difference detection scheme, whose measurement operator can be written as $a_\mathrm{out}^\dagger a_\mathrm{out}-b_\mathrm{out}^\dagger b_\mathrm{out}$. The latter is used most widely for the TMSV state in various quantum spectroscopy and imaging applications^24^ due to strong photon-number correlations between the signal and the idler modes. An unbiased estimator for the coincidence-counting scheme yielding the estimate *T* can be algebraically worked out to be

3 $$\begin{aligned} \hat{T}_\mathrm{Q}^\mathrm{coin}=\frac{a_\mathrm{out}^\dagger a_\mathrm{out}b_\mathrm{out}^\dagger b_\mathrm{out}}{ \eta (2n^2+n)+n n_\mathrm{th}}, \end{aligned}$$

where *n* is the average photon number in each mode. Its expectation value is *T* and its variance is

4 $$\begin{aligned} \langle ( \Delta \hat{T}_\mathrm{Q}^\mathrm{coin} )^2 \rangle&= \frac{Tn}{(\eta (2n^2+n)+n n_\mathrm{th})^2} \Big \{ n_\mathrm{th}^2(3Tn+2)+n_\mathrm{th}[1+4\eta +20T\eta n^2 +2n(T+4\eta +7T\eta )]\nonumber \\&\;\;\;\;+\eta [1+20T\eta n^3 +n(2+4T+4\eta +3T\eta )+n^2(6T+6\eta +20T\eta )] \Big \} . \end{aligned}$$

For the number-difference detection scheme, an unbiased estimator yielding the estimate *T* reads

5 $$\begin{aligned} \hat{T}_\mathrm{Q}^\mathrm{diff}=\frac{a_\mathrm{out}^\dagger a_\mathrm{out}-b_\mathrm{out}^\dagger b_\mathrm{out}+\eta n+n_\mathrm{th}}{n}, \end{aligned}$$

which has the expectation value *T* and the variance

6 $$\begin{aligned} \langle ( \Delta \hat{T}_\mathrm{Q}^\mathrm{diff})^2 \rangle = \big [ (T-\eta )^2 (n^2+n) + n_\mathrm{th}(n_\mathrm{th}+1 + 2\eta n)+T(1-T)n+\eta (1-\eta )n \big ] /n^2. \end{aligned}$$

We are interested in finding the regimes in which one of the quantum schemes outperforms the classical scheme. The performance of a given scheme is quantified by the SNR defined as

7 $$\begin{aligned} \textrm{SNR}= \frac{\langle \hat{T}\rangle }{\sqrt{\langle ( \Delta \hat{T})^2 \rangle }} = \frac{ T}{\sqrt{\langle ( \Delta \hat{T})^2 \rangle }}. \end{aligned}$$

In the following, we compare the performances of the introduced classical and quantum schemes using the SNR as a measure. Then, we compare these schemes with the optimal detection scheme for the chosen states. The optimal SNRs can be obtained using the quantum Cramér-Rao bound for the variance^25–28^: $\langle ( \Delta \hat{T})^2 \rangle \ge 1/H$, where *H* is the quantum Fisher information (QFI). Using a relation between the quantum fidelity and the QFI (see supplementary Information for a quick summary), one can calculate the QFIs for the classical and quantum setups, which can be written as

8 $$\begin{aligned} H_\mathrm{C}=&\frac{\eta n}{T(2n_\mathrm{th}+1)}, \end{aligned}$$

9 $$\begin{aligned} H_\mathrm{Q}=&\frac{n}{T}\left\{ n_\mathrm{th}^2(2Tn+1)+\eta n\left[ 1+\eta n-T(1- \eta +\eta n)\right] \right. \left. +n_\mathrm{th}\left[ 1+T\eta n^2+n(T+2\eta -3T\eta )\right] \right\} / \nonumber \\&\left\{ \left[ n_\mathrm{th}+Tn n_\mathrm{th}+\eta n(1-T)\right] \right. \left. \times \left[ 1+n_\mathrm{th}+2Tn n_\mathrm{th}+n(T+\eta -2T\eta )\right] \right\} , \end{aligned}$$

respectively.

## Results

In this section, we calculate the SNRs for the TMSV state under the coincidence-counting scheme, the number-difference measurement scheme, and the optimal scheme in order to determine the parameter regimes in which the entangled state exhibits an advantage over the coherent state. For the latter, we consider the number counting and optimal schemes.

Since an experimentally generated TMSV state usually has a small mean photon number, we fix the signal strength to $n= 2$ and focus on $n_\mathrm{th} = 0.1,2,4$, i.e., when the thermal noise is weak, comparable to, or larger than the signal strength. It is clear that such a choice of parameters provides rich information about general behaviors of the schemes under investigation. Furthermore, we use $\gamma =1-\eta$, which enables us to explain the estimation performances in terms of the loss in the signal beam.

### The classical scheme

The SNR under the photon counting scheme can be calculated from Eq. (2) and reads

10 $$\begin{aligned} \textrm{SNR}_\mathrm{C} = \frac{T(1-\gamma )n}{\sqrt{T(1-\gamma )n(2n_\mathrm{th}+1) + n_\mathrm{th}(1+n_\mathrm{th})}}. \end{aligned}$$

**Figure 2:**
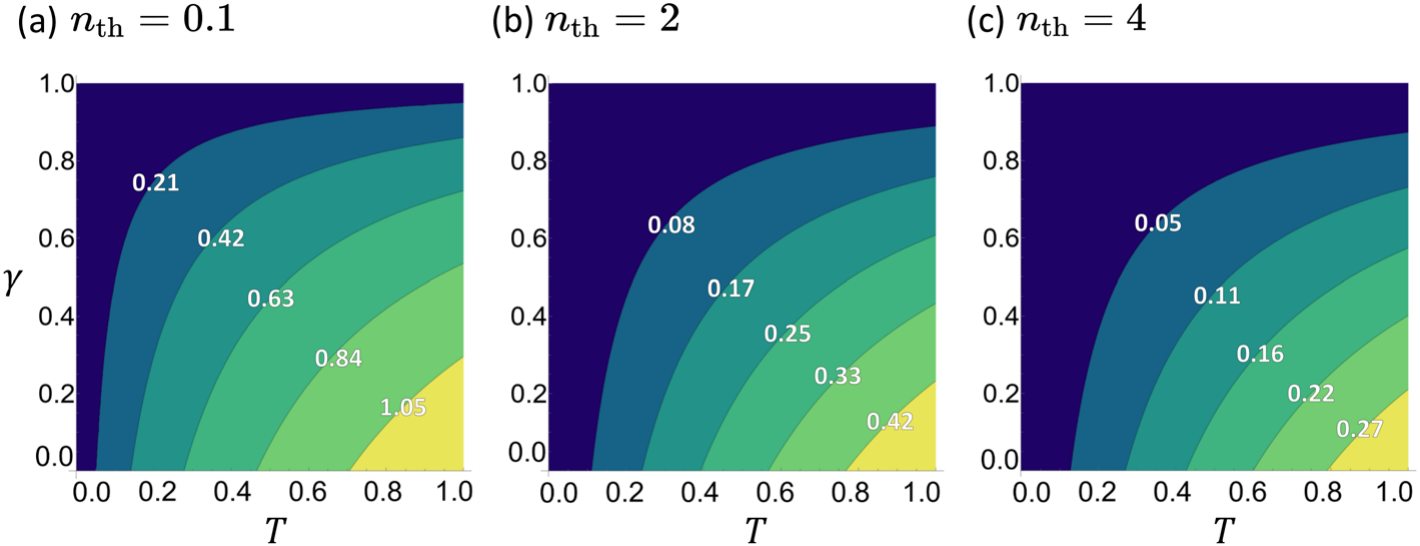
The SNR for the coherent state as a function of $\gamma$ and *T* under photon counting scheme. The average input photon number is $n=2$ and the thermal photon number is $n_\mathrm{th}=0.1 , 2,$ and 4. The SNR increases as (i) *T* increases, (ii) $\gamma$ decreases, and (iii) $n_\mathrm{th}$ decreases.

It is clear that the SNR increases with both *n* and *T*, but decreases with increasing $\gamma$ and $n_\mathrm{th}$. Such behavior is consistent with the experimental result of Ref. ^1^ and is illustrated in Fig. 2.

### The quantum schemes with the thermal noise in the idler mode

In order to examine possible quantum advantages of using the TMSV state, we compare the SNR of the two quantum schemes with the $\text {SNR}_\text {C}$ defined above. The quantum advantage is quantified by the ratio between the SNRs: $\mathrm R_{\kappa } = \text {SNR}_{\kappa }/\text {SNR}_\mathrm{C}$, where $\kappa$ represents a measurement scheme, i.e., ‘coin’ or ‘diff’. $\mathrm R_{\kappa } > 1$ indicates that the quantum scheme $\kappa$ outperforms the classical benchmark, while $\mathrm R_{\kappa } < 1$ indicates that the classical scheme outperforms the quantum schemes.

**Figure 3:**
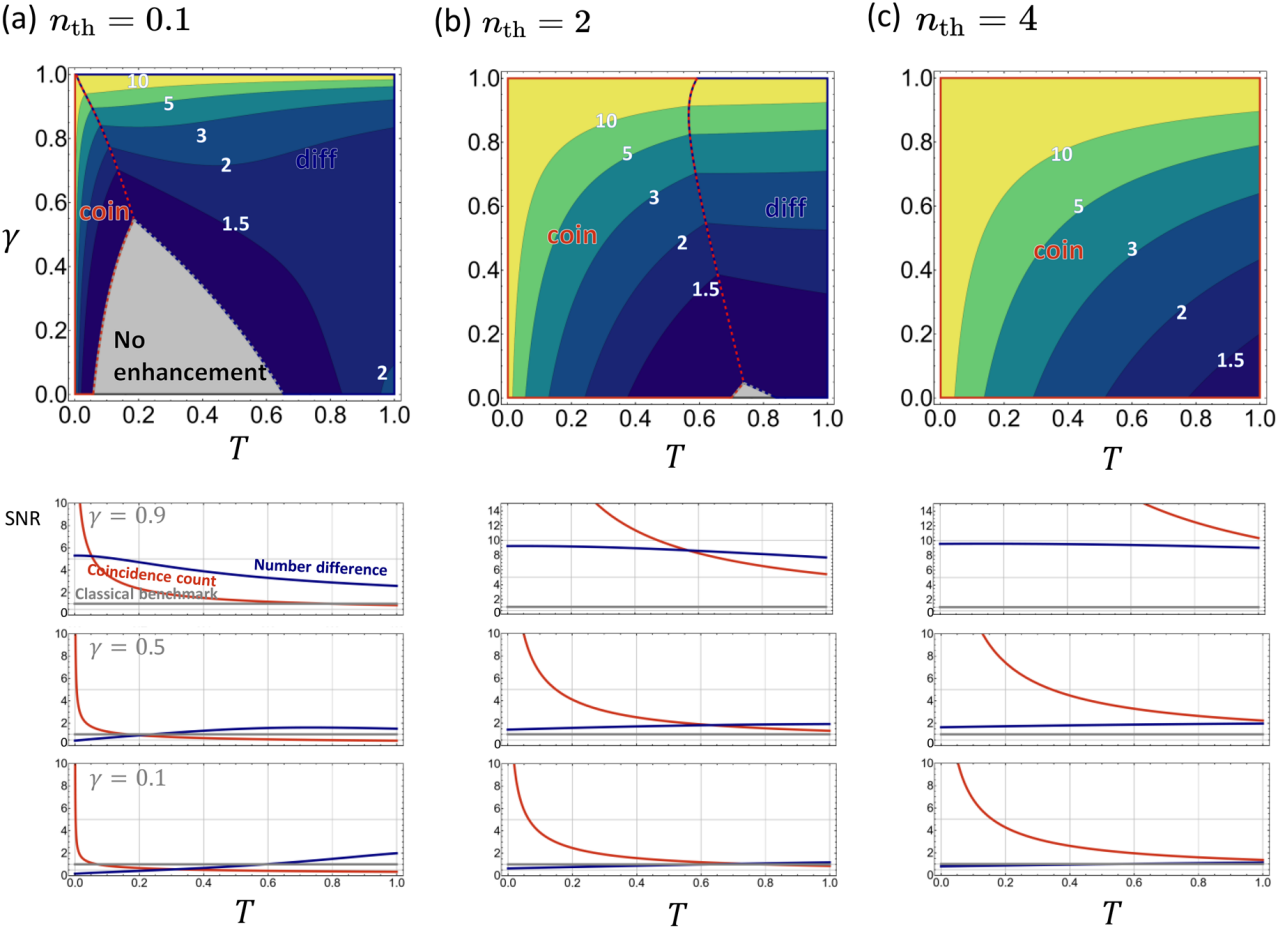
Top row: Quantum enhancement achieved by the TMSV state as quantified by $\mathrm R_\kappa$. The region in which the coincidence-counting scheme outperforms the number-difference and classical schemes is enclosed by the red box and is labeled ‘coin’ while the region dominated by the number-difference scheme is enclosed by the blue box and is labeled ‘diff’. The grey area indicates where no quantum advantage is observed by either schemes. Bottom row: The ratio $\mathrm R_\kappa$ as a function of *T* for $\gamma = 0.1,0.5,$ and 0.9. Note that the coincidence-counting scheme becomes the best choice for all parameter values when $n_\mathrm{th}$ is sufficiently large.

Figure 3 illustrates a comparison among the three schemes. The top row shows the maximum values of $\mathrm R_\kappa$ between the two quantum schemes for $n_\text {th}=0.1, 2, 4$. The comparison reveals three separate regions for given values of $\gamma$, *T*, and $n_\text {th}$. When one of the two quantum schemes outperforms the classical benchmark, the region is labeled by the best scheme $\kappa$, otherwise, the region is labeled ‘No enhancement’. Roughly speaking, the figure shows that the number-difference measurement is the best when the transmitted signal is sufficiently large compared to the thermal noise, while the coincidence-counting scheme works best in the opposite case (except in the region of large $\gamma$, which will be discussed later). Also noteworthy is the fact that the ‘No enhancement’ region occupies a significant portion of the parameter space when the noise is small, but the area shrinks as the thermal noise level increases. On the other hand, the coincidence-counting scheme dominates in almost the entire parameter space when the thermal noise is larger than the signal strength, which is in agreement with the experimental conclusion of Ref. ^1^. The region labeled ‘coin’ further shrinks as $n_\mathrm{th} \rightarrow 0$, but the overall shape remains similar to that of $n_\mathrm{th} = 0.1$, as shown in Supplementary Fig. S1. Plots of $\mathrm R_{\kappa}$ provide useful information on quantum advantages but not on the actual sensitivities achieved by the quantum schemes. The latter is provided in Supplementary Fig. S2, which plots $\Delta T$ as a function of $\gamma$ and *T*.

The dependence of $\mathrm R_\kappa$ on *T* for specific values of $\gamma$ is more clearly shown on the bottom row of Fig. 3. In the quantum setup shown in Fig. 1b, the loss parameter $\gamma$ determines how much of the idler beam is lost while the mean number of thermal photons entering the detector is fixed to $n_\text {th}$. For a small value of $n_\mathrm{th}$, $\mathrm R_\mathrm{coin}$ diverges as $T \rightarrow 0$, making the coincidence-counting scheme the best option when the sample transmittance is very small, i.e., $T\ll 1$, whereas the number-difference scheme is the best when $T\approx 1$. For a sufficiently large value of $n_\mathrm{th}$, on the other hand, the coincidence-counting scheme outperforms the other two schemes for all values of *T*. Interestingly, the observed quantum advantages increase as either the loss or the thermal noise level increases, i.e., as $\gamma$ or $n_\text {th}$ increases. This is because it is the idler mode that suffers from the imperfections in the quantum scheme (Fig. 1b), while it is the signal mode that suffers from them in the classical scheme (Fig. 1a). This motivates a further investigation on an alternative quantum scheme shown in Fig. 5 as will be discussed later.

### The optimal detection scheme

As discussed in the previous section, the SNR depends on the chosen detection scheme, for a given input state. It means that the SNR can be maximized over all possible detection schemes, leading to the optimal $\text {SNR}^\text {opt}=T\sqrt{H}$, given by the quantum Cramér–Rao bound for the QFI *H*. For the coherent state input, one obtains $\textrm{SNR}_\mathrm{C}^\mathrm{opt} = \sqrt{T(1-\gamma )n}/(2n_\mathrm{th}+1)$, while for the two-mode squeezed vacuum state input, $\text {SNR}_\text{Q}^{\text{opt}}$ can be obtained straightforwardly from Eq. (9) but is too cumbersome to write here. Incidentally, the classical scheme is optimal for $n_\mathrm{th} = 0$, as can be verified by comparing $\textrm{SNR}_\mathrm{C}^\mathrm{opt}$ with Eq. (10).

**Figure 4:**
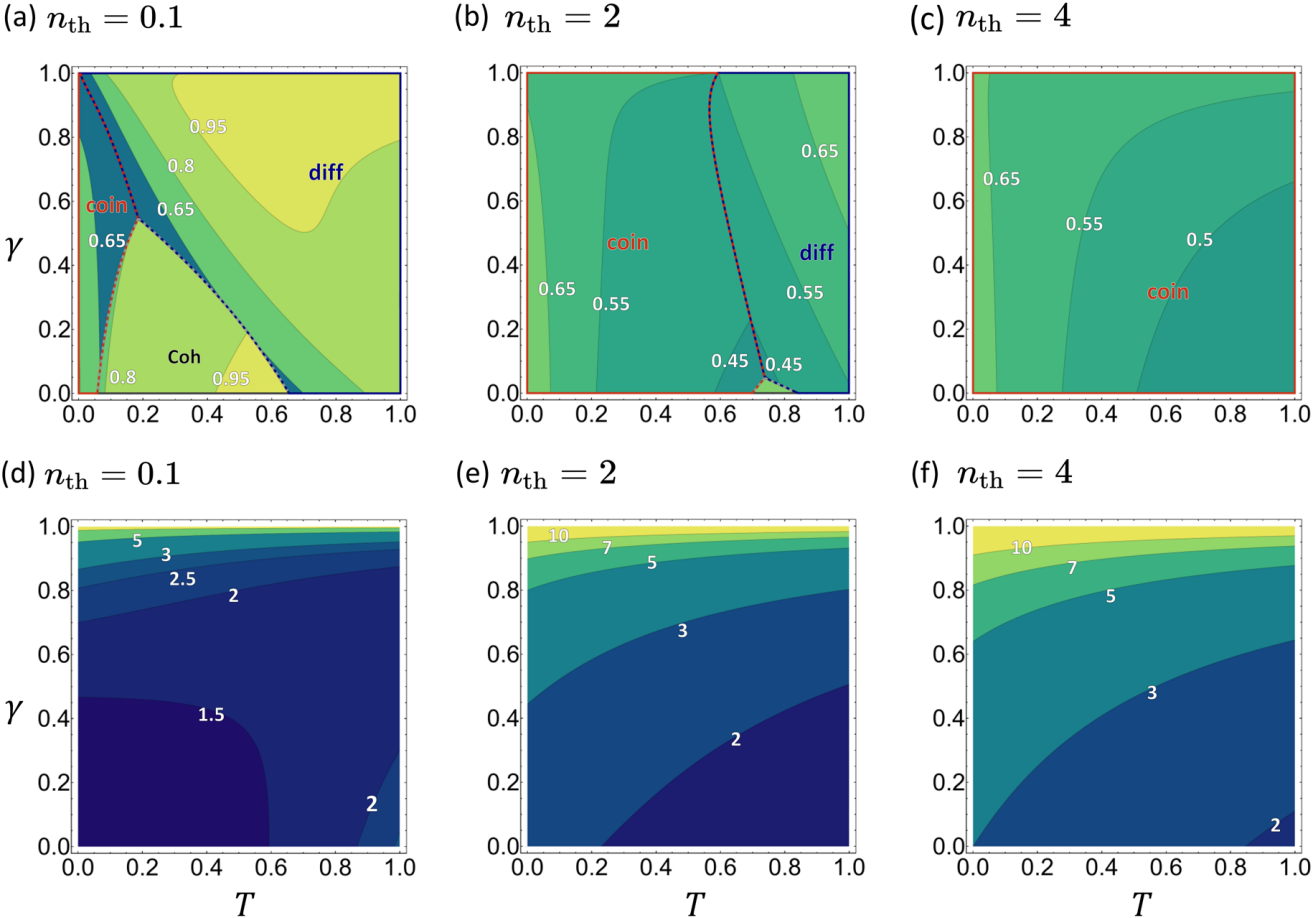
Top row: $\mathrm R_\kappa ^\mathrm{opt}$ of the measurement schemes in their respective regions of dominance. Bottom row: $\mathrm R_\mathrm{QFI}$, illustrating the upper bound on achievable quantum enhancement.

Let us now compare the optimal SNRs with the SNRs calculated in the previous section in order to examine the optimality of the considered detection schemes. To this end, we define the ratios $\mathrm R_\mathrm{Coh}^\mathrm{opt} = \text {SNR}_\text {C}/\text {SNR}_\text {C}^\text {opt}$ and $\mathrm R_\kappa ^\mathrm{opt} = \text {SNR}_\kappa /\text {SNR}_\text {Q}^\text {opt}$ for the classical and quantum schemes, respectively. The upper panels of Fig. 4 illustrate the ratios. In each plot, the regions are divided in accordance with Fig. 3. Interestingly, the number-difference scheme and the classical scheme are nearly optimal in a wide region of the parameter space when $n_\mathrm{th}$ is small, but the coincidence-counting scheme is only sub-optimal. All three schemes become less optimal with increasing $n_\mathrm{th}$ however, until the coincidence-counting scheme eventually dominates the entire parameter space.

Next, we compare the optimal SNRs of the coherent state and the TMSV state, as quantified by the ratio $\mathrm R_\mathrm{QFI} = \text {SNR}_\text{Q}^{\text{opt}}/\text {SNR}_\text{ C}^{\text{opt}}$. The bottom panels of Fig. 4 plots $\mathrm R_\mathrm{QFI}$. They clearly show that the optimum quantum scheme always outperforms any classical scheme regardless of the values of *T* and $\gamma$. Most remarkably, the quantum advantage increases with increase in either the thermal photon number $n_\mathrm{th}$ or the loss rate $\gamma$. That is, the larger the environmental noise, the larger the quantum advantage observed. In fact, the ratio $\mathrm R_\text {QFI}$ goes as ${2n_\mathrm{th}}/{(Tn+1)(1-\gamma )}$, as $n_\text {th} \rightarrow \infty$.

## Alternative quantum setup with the thermal noise in the signal mode

From the above considerations, we are led to conclude that the quantum schemes significantly outperform the classical schemes even when the thermal noise dominates. This sounds interesting at first, but puzzling on a second thought since quantum features are typically destroyed by noise and loss, leaving classical schemes as preferred options in noisy and lossy environments. Looking back to Fig. 1, it is not too difficult to see a potential source of this counter-intuitive behavior. In the classical setup, thermal noise is added to the same mode that goes through the sample, whereas in the quantum setup, it is added to the idler mode which is only used as a reference. Therefore, in the extreme case in which the noise intensity is much larger than that of the source, one would benefit by simply discarding the idler mode in the quantum scheme, while there is no such option in the classical scheme. This renders the quantum schemes more advantageous as the noise and loss increases.

**Figure 5:**
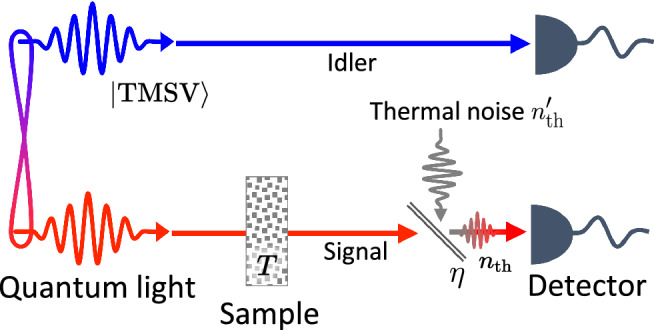
Schematic illustration of an alternative quantum setup, in which the noise is introduced to the signal mode.

To make a fairer comparison with the classical scheme we revise the previous quantum setup so that the thermal noise enters the signal mode, as depicted in Fig. 5. In the new quantum setup, the idler mode no longer experiences any thermal noise. The signal mode, on the other hand, experiences a thermal noise with an average photon number $n_\mathrm{th}$ after passing through the sample.

As before, the thermal noise can be modeled as a beam splitter of transmittance $\eta$ where a thermal field with the average photon number $n_\mathrm{th}/(1-\eta )$ is fed into the unused port. At this point, the setup can be simplified by merging the two beam splitters into a single beam splitter of transmittance $T' \equiv T\eta$ in which the thermal field, entering the unused port of the beam splitter, has the average photon number $n_\mathrm{th}' = n_\mathrm{th}/(1-T\eta )$. The beam splitters in the classical scheme can be merged in the same way. In the new number-difference scheme, the variance for *T* can be written as

11 $$\begin{aligned} \langle (\Delta \hat{T}_\mathrm{NQS}^\mathrm{diff})^2\rangle =&\big [ n^2(T(1-\gamma )-1)^2 +T(1-\gamma )n(2n_\mathrm{th}-1)+ n + n_\mathrm{th}(n_\mathrm{th}+1)\big ] /((1-\gamma )n)^2, \end{aligned}$$

which is clearly a function of $T'$. Similarly, the variance for the coincidence-counting scheme depends on $T'$ but the complete expression is too cumbersome to write down here.

### Quantum enhancement

**Figure 6:**
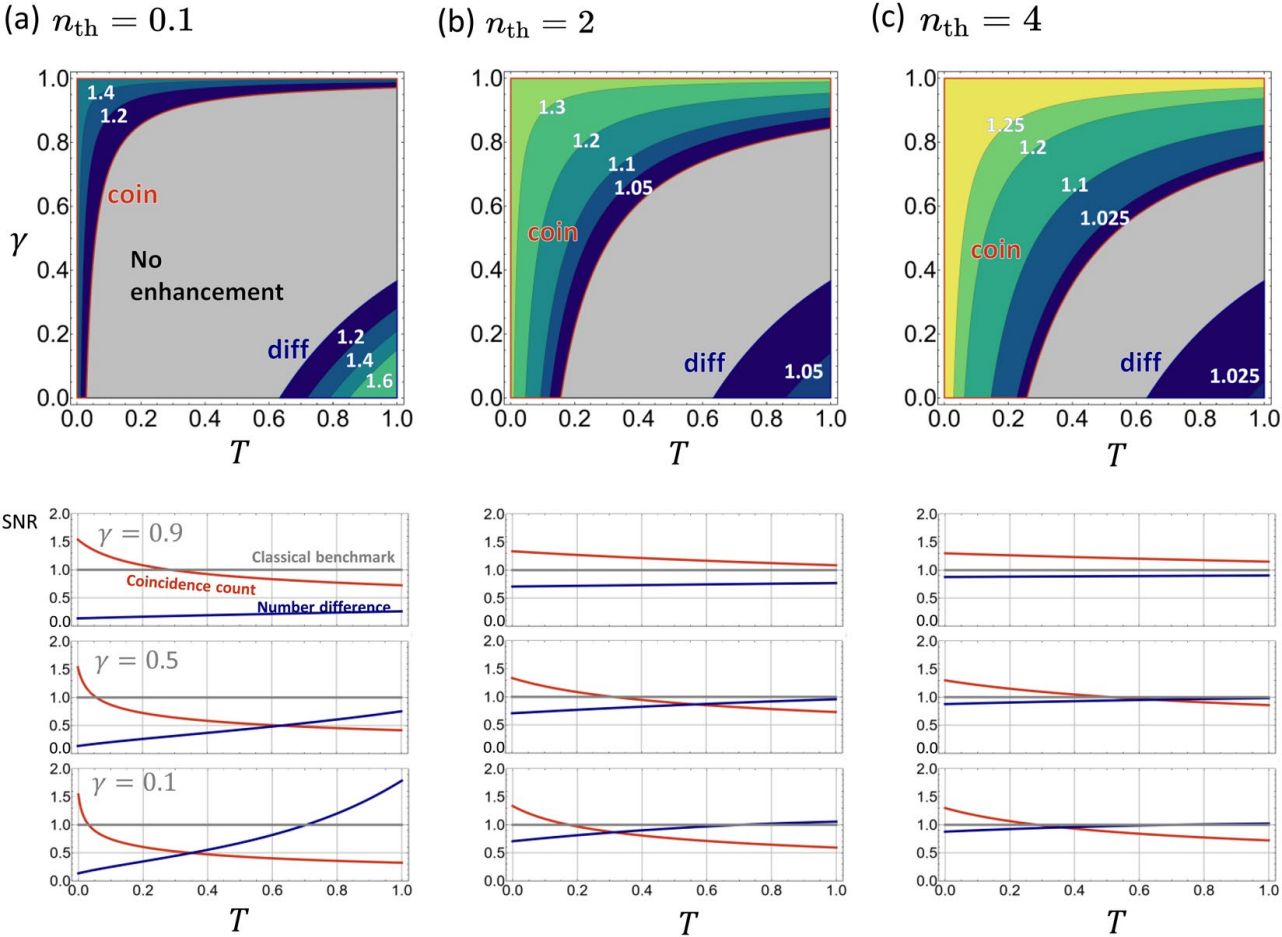
Quantum enhancement achieved by the TMSV state as quantified by $\mathrm R_\kappa$ in the new quantum setup. The regions are labeled in the same way as in Fig. 3. Note the diminished quantum enhancement compared to Fig. 3.

Quantum enhancements in the new quantum schemes are depicted in Fig. 6 (also see Supplementary Fig. S3 for plots of $\Delta T$). A quick comparison with Fig. 3 shows that the quantum enhancements are strongly suppressed in the new setup. The obvious symmetry in the $(\gamma , T)$ space stems from the mentioned dependence on the total signal loss $T' = T\eta$. When $n_\text {th}$ is small, quantum schemes are better in the two extreme limits of large or small total loss. For large total loss, i.e., $T' \approx 0$, the coincidence-counting scheme beats both the classical and number-difference schemes. In the opposite limit of small total loss ($T' \approx 1$), the number-difference scheme is the best choice.

As $n_\mathrm{th}$ is decreased further from $n_\mathrm{th} = 0.1$, the coincidence counting scheme loses its advantage over the classical scheme and $\mathrm R_\mathrm{coin} \rightarrow 1$ as $n_\text {th} \rightarrow 0$. The number-difference scheme, on the other hand, keeps its advantage in roughly the same parameter region as for $n_\mathrm{th} = 0.1$, but with increased values of $\mathrm R_\mathrm{diff}$. The behavior at $n_\mathrm{th}=0$ is shown in Supplementary Fig. S1. With increasing values of $n_\text {th}$, the coincidence-counting scheme starts to dominate the parameter space until it covers the entire parameter space in the $n_\text {th} \rightarrow \infty$ limit (not shown). One obtains a simple formula $\mathrm R_\mathrm{coin} \rightarrow (2n+1)^2/(3n^2+2n)$ in this limit, which is a monotonically decreasing function of *n*. It shows that there is a tremendous quantum enhancement for small *n* and a minimum of 33.33% advantage, i.e. $\mathrm R_\mathrm{coin}>4/3$. On the other hand, the number-difference scheme becomes less and less effective, until its advantage over the classical scheme is completely lost. In fact, $\mathrm R_\mathrm{diff} \rightarrow 1$ in the limit of infinite thermal photon number.

### The optimal detection scheme

**Figure 7:**
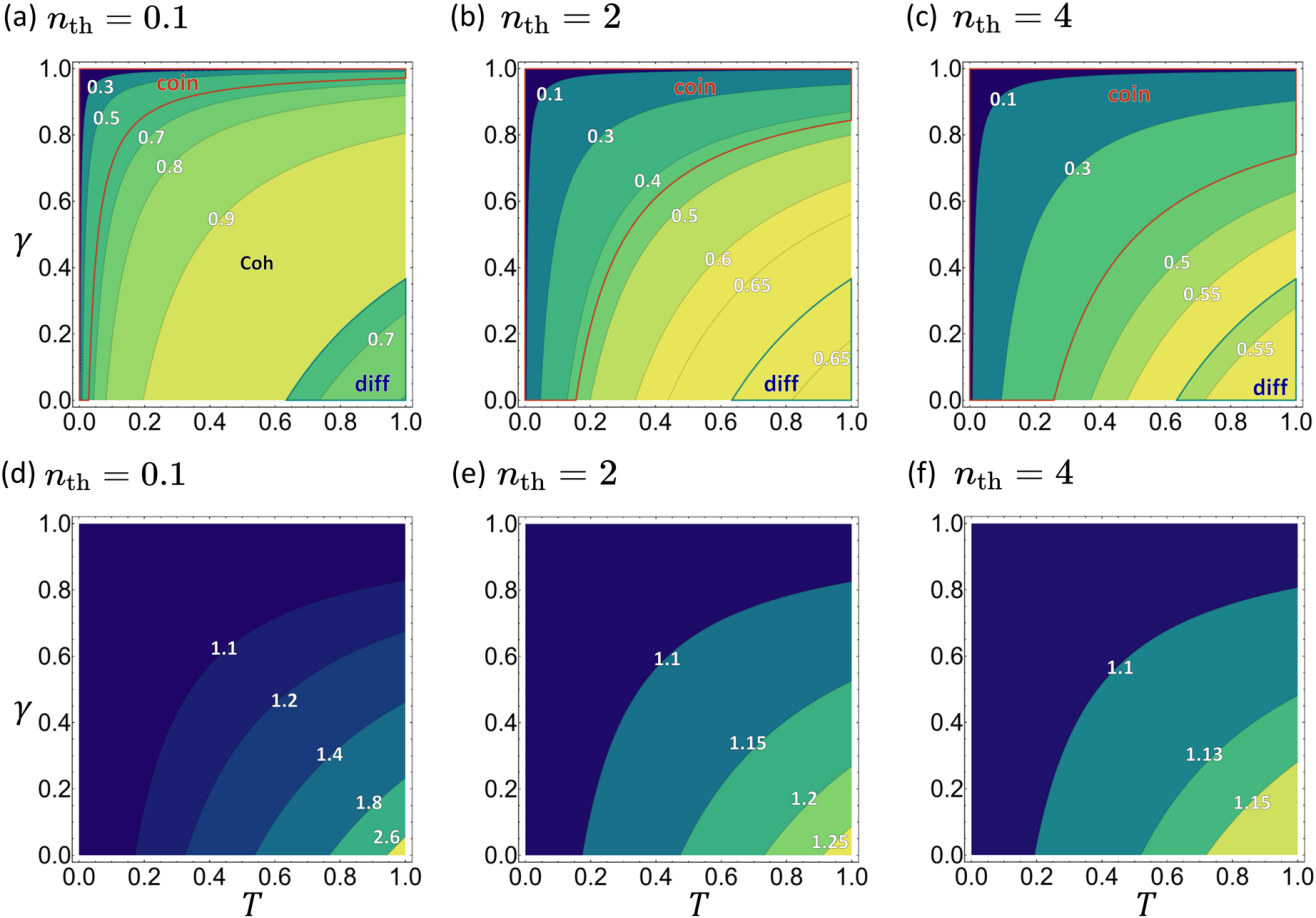
Top row: $\mathrm R_\kappa ^\mathrm{opt}$ in their respective regions of dominance, for the alternative setup. Bottom row: $\mathrm R_\mathrm{QFI}$, illustrating the upper bound on the achievable quantum enhancement.

A comparison between the chosen measurement schemes and the optimal schemes is displayed in the upper row of Fig. 7, where $\mathrm R_\kappa ^\mathrm{opt}$s are plotted in regions classified according to Fig. 6. Compared to the original setup (see Fig. 4) we note that: (i) the two quantum schemes have smaller regions of dominance and subdued enhancements over the optimum quantum scheme; (ii) the optimality of all three schemes increases as $T'$ increases; (iii) the optimality of all three schemes increases as $n_\mathrm{th}$ decreases.

The bottom row of Fig. 7 illustrates the achievable quantum advantage as quantified by $\mathrm R_\mathrm{QFI}$. The ratio $\mathrm R_\mathrm{QFI} = \text{SNR}_\text{Q}^{\text{opt}}/\text{SNR}_\text{C}^{\text{opt}}$ increases with increasing $T'$ and decreasing $n_\mathrm{th}$. Although quantum advantages are more moderate compared to those for the original setup (see Fig. 4), they persist for all parameter regimes. When $n_\text {th}$ is small, greater than two-fold enhancement is achieved when $T' \approx 1$. In fact $\mathrm{R_{QFI}} \rightarrow (1-T')^{-1/2}$ as $n_\mathrm{th} \rightarrow 0$, indicating that the quantum advantages, as quantified by $\mathrm R_{\text{QFI}}$, goes to infinity. Note that the dependence on the signal photon number *n* disappears in this limit. In the opposite limit of $n_\text {th}\rightarrow \infty$, the quantum advantage becomes independent of *T* and $\gamma$: $\mathrm{R_{QFI}} = [2(n+1)/(2n+1)]^{1/2}$. Interestingly, quantum advantage persists even in the presence of strong thermal background and is more significant for smaller values of *n*. For the chosen value of $n=2$, $\mathrm R_\mathrm{QFI} \approx 1.1$, i.e., a 10% advantage.

## Summary and discussion

We have theoretically analyzed the experimental quantum spectroscopy setup presented in Ref. ^1^. Our calculations confirm the observed quantum advantages of the TMSV state under the coincidence-counting scheme when the thermal noise is strong. We have also studied the number-difference scheme and showed that it exhibits quantum advantages over a wide region in the $(\gamma , T)$ parameter space when the thermal noise is weak. In the latter case, we also showed that the classical scheme outperforms both the number-difference and coincidence-counting schemes in a significant region of the parameter space.

We then compared the signal-to-noise ratios under the optimal classical and quantum schemes by calculating the QFI, which revealed that a significant amount of quantum enhancement is possible. Noting that this counter-intuitive behavior stems from the asymmetric way in which the noise is introduced in the classical and quantum setups, we moved on to introduce an alternative quantum setup in which the noise is introduced to the signal mode. The quantum enhancements of the number-difference and coincidence-counting schemes survive in the new setup, but are strongly suppressed compared to the original setup. We showed, however, that quantum advantages persist under an optimum measurement scheme.

An important future research direction is to find out the best implementable schemes. For example, a quick inspection of Figs. 6c and 7c demonstrates that the coincidence counting scheme is far from being optimal but still exhibits a significant quantum advantage compared to the chosen classical scheme. Figure 7f then suggests that not only the quantum scheme, but the classical scheme is also sub-optimum. It is therefore of interest to find better implementable classical schemes and compare the performances of the quantum schemes against them. One possible option is to split a thermal light into two modes ^29^, and investigate the performances of coincidence-counting or number-difference detection schemes. Finally, a more straightforward direction is to find the effects of thermal noise introduced to the idler mode. How robust are the schemes against such a noise? We are currently investigating this in detail.

## Supplementary Information

Supplementary Information.

## Data Availability

The datasets used and/or analysed during the current study available from the corresponding author on reasonable request.
