# Supplementary material for: Quantum loss sensing with two-mode squeezed vacuum state under noisy and lossy environment
Source: Sci Rep. 2023 Apr 12;13:5936. doi: 10.1038/s41598-023-32770-7 (PMC10097776; doi:10.1038/s41598-023-32770-7)
Supplement: Supplementary file 1 — Supplementary Information. [file 41598_2023_32770_MOESM1_ESM.pdf]

Quantum loss sensing with two-mode squeezed vacuum state under noisy and lossy environment–Supplementary Information

Sang-il Park and Changsuk Noh
Kyungpook National University, Daegu 41566, Korea

Changhyoup Lee
Korea Research Institute of Standards and Science, Daejeon 34113, Korea

I. QUANTUM FISHER INFORMATION FOR GAUSSIAN STATES

The quantum Fisher information (QFI) can be calculated from the fidelity $\mathcal{F}(\rho_T, \rho_{T+dT})$ between the two states ρ_T and ρ_{T+dT} and is given by [1]

$$H(T) = \frac{8[1-\mathcal{F}(\rho_T, \rho_{T+dT})]}{dT^2}. \tag{1}$$

There is a well-known method of computing the fidelity of the Gaussian states [2]. For n bosonic modes described by quadrature operators $\mathbf{Q} = (x_1, \cdots, x_n, p_1, \cdots, p_n)^T$, the canonical commutation relations can be written as [3]

$$[\mathbf{Q}, \mathbf{Q}^T] = i\Omega_n,\ \Omega_n := \begin{pmatrix} 0_n & I_n \\ -I_n & 0_n \end{pmatrix} \tag{2}$$

where I_n and 0_n are the $n \times n$ identity matrix and null matrix, respectively. The first- and second-order moments of Gaussian states can be written in terms of the mean $(\mathbf{u})$ and the covariance matrix (V) as follows:

$$u_i = \langle Q_i \rangle, \tag{3}$$

$$V_{ij} = \frac{1}{2}\langle \{Q_i - u_i, Q_j - u_j\}\rangle. \tag{4}$$

Using a modified version of the covariance matrix $W := -2Vi\Omega_n$, the fidelity between two Gaussian states is given by

$$\mathcal{F}(\rho_1, \rho_2) = \mathcal{F}_0(V_1, V_2) \exp\left[-\frac{1}{4}\delta_u^T (V_1 + V_2)^{-1}\delta_u\right], \tag{5}$$

where $\delta_u := u_2 - u_1$ and $\mathcal{F}_0(V_1, V_2)$ is given by [2, 4, 5].

$$\mathcal{F}_0^2(V_1, V_2) = \frac{1}{\sqrt{\Delta + \Lambda} - \sqrt{\Lambda}}, \tag{6}$$

for single-mode Gaussian states and

$$\mathcal{F}_0^2(V_1, V_2) = \frac{1}{\sqrt{\Gamma} + \sqrt{\Lambda} - \sqrt{(\sqrt{\Gamma} + \sqrt{\Lambda})^2 - \Delta}}, \tag{7}$$

for two-mode Gaussian states. Here, the symplectic invariants are defined as $\Delta = \det(V_1 + V_2)$, $\Gamma = 2^{2n}\det(\Omega_n V_1 \Omega_n V_2 - I/4)$, and $\Lambda = 2^{2n}\det(V_1 + i\Omega_n/2)\det(V_2 + i\Omega_n 2)$.

The first- and second-order moments of the classical and quantum schemes are given as

$$u_C = \begin{pmatrix} a\sqrt{2T\eta} & b\sqrt{2T\eta} \end{pmatrix}, \quad V_C = \begin{pmatrix} n_{\rm th} + \frac{1}{2} & 0 \\ 0 & n_{\rm th} + \frac{1}{2} \end{pmatrix}, \tag{8}$$

$$u_Q = \begin{pmatrix} 0 & 0 & 0 & 0 \end{pmatrix}, \quad V_Q = \begin{pmatrix} Tn + \frac{1}{2} & \sqrt{T\eta n(n+1)} & 0 & 0 \\ \sqrt{T\eta n(n+1)} & \eta n + n_{\rm th} + \frac{1}{2} & 0 & 0 \\ 0 & 0 & Tn + \frac{1}{2} & -\sqrt{T\eta n(n+1)} \\ 0 & 0 & -\sqrt{T\eta n(n+1)} & \eta n + n_{\rm th} + \frac{1}{2} \end{pmatrix}, \tag{9}$$

where a and b are the complex components of the displacement parameter $\alpha = \sqrt{n}$. Note that n here denotes the mean signal photon number. Now, one can compute the quantum Fisher information of the classical and quantum schemes using Eqs. (1) $\sim$ (9).

A. Outline of the calculation procedure for QFI

Let us start with the classical scheme for the original setup. To calculate the QFI, we use Eq. (5) to calculate the fidelity first, then use Eq. (1). Wtih $\rho_1 = \rho_T$ and $\rho_2 = \rho_{T+dT}$, the first- and second-order moments are readily obtained as

$$u_1 = \left(a\sqrt{2T\eta}\;\; b\sqrt{2T\eta}\right), \quad V_1 = \begin{pmatrix} n_{\rm th}+\frac{1}{2} & 0 \\ 0 & n_{\rm th}+\frac{1}{2} \end{pmatrix}, \tag{10}$$

$$u_2 = \left(a\sqrt{2(T+dT)\eta}\;\; b\sqrt{2(T+dT)\eta}\right), \quad V_2 = \begin{pmatrix} n_{\rm th}+\frac{1}{2} & 0 \\ 0 & n_{\rm th}+\frac{1}{2} \end{pmatrix}. \tag{11}$$

Equation (6) gives $\mathcal{F}_0(V_1, V_2) = 1$ and from Eq. (5) we obtain

$$\mathcal{F}(\rho_1,\rho_2) = \exp\left[\frac{n\left(-\eta dT - 2T\eta + 2\eta\sqrt{T(T+dT)}\right)}{2(2n_{\rm th}+1)}\right]. \tag{12}$$

To obtain the QFI, we first Talyor expand Eq. (12) about dT to obtain

$$1 - \frac{n\eta dT^2}{8(T(2n_{\rm th}+1)} + \frac{n\eta dT^3}{16T^2(2n_{\rm th}+1)} + O[dT]^4, \tag{13}$$

and substitute it into Eq. (1). This yields the QFI of the classical scheme for the original setup:

$$H_{\rm C} = \frac{\eta n}{T(2n_{\rm th}+1)} \tag{14}$$

.

The procedure is more or less the same in the quantum scheme, but with different first and second-order moments. We also need to use Eq. (7) instead of Eq. (6) because we are dealing with a two-mode state. Because the first order moment u_Q is zero the exponential term in Eq. (5) is 1, and all we need is the $\mathcal{F}_0$ term. The latter is too cumbersome to write down, but nevertheless calculable with the aid of a symbolic programming language form which the QFI can be calculated in the same way as above. The QFIs for the alternative setup are calculated in the same fashion, but with different density operator ρ_T, hence different first and second-order moments.

II. UNBIASED ESTIMATORS AND VARIANCES

Here we outline the procedures for (i) obtaining the unbiased estimators used in the main text and (ii) calculations of variances. Because the procedure is the same for the original and alternative setups, we will only provide outlines for the original setup.

A. Classical scheme

The initial state of the classical scheme is $\rho_{\rm in} = \rho_{\rm coh} \otimes \rho_{\rm vac} \otimes \rho_{n'_{\rm th}}$, where $\rho_{\rm coh}$, $\rho_{\rm vac}$, and $\rho_{n'_{\rm th}}$ represent the input coherent, vacuum, and thermal states, respectively. The bosonic operator of the output state is given by

$$a_{\rm out} = \sqrt{T\eta}a_{\rm in} + \sqrt{\eta(1-T)}c_{\rm in} + \sqrt{1-\eta}d_{\rm in}, \tag{15}$$

where $a_{\rm in}$, $c_{\rm in}$, and $d_{\rm in}$ are the annihilation operators of the input coherent, vacuum, and thermal states, respectively and T and η are the transmittances of the sample and the noisy environment, respectively. To obtain an unbiased estimator, we calculate the expectation value for the photon number counting measurement:

$$\begin{aligned}
{\rm tr}[a^\dagger_{\rm out}a_{\rm out}\rho_{\rm out}] =& {\rm tr}[[(T\eta a^\dagger_{\rm in}a_{\rm in} + \sqrt{T(1-T)\eta^2}(a^\dagger_{\rm in}c_{\rm in} + a_{\rm in}c^\dagger_{\rm in}) + \sqrt{T\eta(1-\eta)}(a^\dagger_{\rm in}d_{\rm in} + a_{\rm in}d^\dagger_{\rm in}) \\
&+ \sqrt{(1-T)\eta(1-\eta)}(c^\dagger_{\rm in}d_{\rm in} + c_{\rm in}d^\dagger_{\rm in}) + \eta(1-T)c^\dagger_{\rm in}c_{\rm in} + (1-\eta)d^\dagger_{\rm in}d_{\rm in})\rho_{\rm in}] \\
=& T\eta n + (1-\eta)n'_{\rm th} \\
=& T\eta n + n_{\rm th}.
\end{aligned} \tag{16}$$

From which one readily obtains an unbiased estimator $\hat{T}_C = \frac{a_{\text{out}}^\dagger a_{\text{out}} - n_{\text{th}}}{\eta n}$. The variance of the unbiased estimator is then

$$\begin{aligned}\text{tr}[[(\Delta \hat{T}_C)^2 \rho_{\text{out}}] =&\text{tr}[[(\frac{a_{\text{out}}^\dagger a_{\text{out}} - n_{\text{th}}}{\eta n})^2 \rho_{\text{out}}] - \text{tr}[[(\frac{a_{\text{out}}^\dagger a_{\text{out}} - n_{\text{th}}}{\eta n})\rho_{\text{out}}]^2 \\ =&\frac{T(2n_{\text{th}}+1) + \frac{n_{\text{th}}(n_{\text{th}}+1)}{\eta n}}{\eta n}. \end{aligned} \tag{17}$$

B. Quantum schemes

In the quantum case, the initial state of the quantum scheme is changed to $\rho_{\text{in}} = \rho_{\text{tmsv}} \otimes \rho_{\text{vac}} \otimes \rho_{n'_{\text{th}}}$, where ρ_{tmsv}, ρ_{vac}, and $\rho_{n'_{\text{th}}}$ represent the input two-mode squeezed vacuum state, the vacuum (for the sample), and thermal states, respectively. Annihilation operators of the output modes are given by

$$a_{\text{out}} = \sqrt{T} a_{\text{in}} + \sqrt{1-T} c_{\text{in}}, \tag{18}$$

$$b_{\text{out}} = \sqrt{\eta} b_{\text{in}} + \sqrt{1-\eta} d_{\text{in}}, \tag{19}$$

where a_{in}, b_{in} are the annihilation operators of the two-mode squeezed vacuum state and c_{in}, and d_{in} are the annihilation operators of the vacuum state and thermal state, respectively. For the photon number difference measurement, the average is

$$\begin{aligned}\text{tr}[(a_{\text{out}}^\dagger a_{\text{out}} - b_{\text{out}}^\dagger b_{\text{out}})\rho_{\text{out}}] =&\text{tr}[(T a_{\text{in}}^\dagger a_{\text{in}} + \sqrt{T(1-T)}(a_{\text{in}}^\dagger c_{\text{in}} + a_{\text{in}} c_{\text{in}}^\dagger) + (1-T) c_{\text{in}}^\dagger c_{\text{in}} \\ &- \eta b_{\text{in}}^\dagger b_{\text{in}} - \sqrt{\eta(1-\eta)}(b_{\text{in}}^\dagger d_{\text{in}} + b_{\text{in}} d_{\text{in}}^\dagger) - (1-\eta) d_{\text{in}}^\dagger d_{\text{in}})\rho_{\text{in}}] \\ =&Tn - \eta n - (1-\eta) n'_{\text{th}} \\ =&Tn - \eta n - n_{\text{th}}, \end{aligned} \tag{20}$$

from which an unbiased estimator is readily obtained as, $\hat{T}_{\text{ND}} = \frac{(a_{\text{out}}^\dagger a_{\text{out}} - b_{\text{out}}^\dagger b_{\text{out}}) + \eta n + n_{\text{th}}}{n}$. The variance of the unbiased estimator is then

$$\begin{aligned}\text{tr}[(\Delta \hat{T}_{\text{ND}})^2 \rho_{\text{out}}] =&\text{tr}[(\frac{(a_{\text{out}}^\dagger a_{\text{out}} - b_{\text{out}}^\dagger b_{\text{out}}) + \eta n + n_{\text{th}}}{n})^2 \rho_{\text{out}}] - \text{tr}[(\frac{(a_{\text{out}}^\dagger a_{\text{out}} - b_{\text{out}}^\dagger b_{\text{out}}) + \eta n + n_{\text{th}}}{n})\rho_{\text{out}}]^2 \\ =&\frac{(T-\eta)^2(n^2+n) + n_{\text{th}}(2\eta n + n_{\text{th}} + 1) + T(1-T)n + \eta(1-\eta)n}{n^2}. \end{aligned} \tag{21}$$

The operator for the coincidence counting scheme is given by $a_{\text{out}}^\dagger a_{\text{out}} b_{\text{out}}^\dagger b_{\text{out}}$. The expectation value is

$$\begin{aligned}\text{tr}[(a_{\text{out}}^\dagger a_{\text{out}} b_{\text{out}}^\dagger b_{\text{out}})\rho_{\text{out}}] =&\text{tr}[(T a_{\text{in}}^\dagger a_{\text{in}} + \sqrt{T(1-T)}(a_{\text{in}}^\dagger c_{\text{in}} + a_{\text{in}} c_{\text{in}}^\dagger) + (1-T) c_{\text{in}}^\dagger c_{\text{in}})(\eta b_{\text{in}}^\dagger b_{\text{in}} \\ &+ \sqrt{\eta(1-\eta)}(b_{\text{in}}^\dagger d_{\text{in}} + b_{\text{in}} d_{\text{in}}^\dagger) + (1-\eta) d_{\text{in}}^\dagger d_{\text{in}})\rho_{\text{in}}] \\ =&T\eta(2n^2+n) + T(1-\eta) n n'_{\text{th}} \\ =&T(\eta(2n^2+n) + n n_{\text{th}}), \end{aligned} \tag{22}$$

which yields an unbiased estimator

$$\hat{T}_{\text{CC}} = \frac{(a_{\text{out}}^\dagger a_{\text{out}} b_{\text{out}}^\dagger b_{\text{out}})}{\eta(2n^2+n) + n n_{\text{th}}}. \tag{23}$$

The variance of the unbiased estimator is

$$\begin{aligned}\text{tr}[(\Delta \hat{T}_{\text{CC}})^2 \rho_{\text{out}}] =&\text{tr}[(\frac{(a_{\text{out}}^\dagger a_{\text{out}} b_{\text{out}}^\dagger b_{\text{out}})}{\eta(2n^2+n) + n n_{\text{th}}})^2 \rho_{\text{in}}] - \text{tr}[(\frac{(a_{\text{out}}^\dagger a_{\text{out}} b_{\text{out}}^\dagger b_{\text{out}})}{\eta(2n^2+n) + n n_{\text{th}}})\rho_{\text{in}}]^2 \\ =&\frac{Tn}{(\eta(2n^2+n) + n n_{\text{th}})^2}\Big\{ n_{\text{th}}^2(3Tn+2) + n_{\text{th}}[1 + 4\eta + 20T\eta n^2 + 2n(T + 4\eta + 7T\eta)] \\ &+ \eta[1 + 20T\eta n^3 + n(2 + 4T + 4\eta + 3T\eta) + n^2(6T + 6\eta + 20T\eta)]\Big\}. \end{aligned} \tag{24}$$

III. QUANTUM ENHANCEMENTS FOR $n_{\text{th}} = 0$

Figures S1(a) and (b) depict how Figs. 3 and 5 in the main text change, respectively, in the limit of vanishing thermal photon number, i.e., $n_{\text{th}} \to 0$. For the original asymmetric setup (Fig. S1(a)), the 'No enhancement' region occupies a larger parameter space at the cost of reduced region for 'coin', while the region occupied by 'diff' stays more or less the same. The values of R_{coin} have decreased significantly from those at $n_{\text{th}} = 0.1$, while R_{diff} exhibits mixed behavior. The latter has increased when T is large and γ is small (bottom right corner), but has decreased when γ is large (for all T). The situation is similar for the alternative quantum setup, as shown in Fig. S1(b), except for the fact that the coincidence-counting scheme exhibits no quantum enhancement at all when $n_{\text{th}} = 0$.

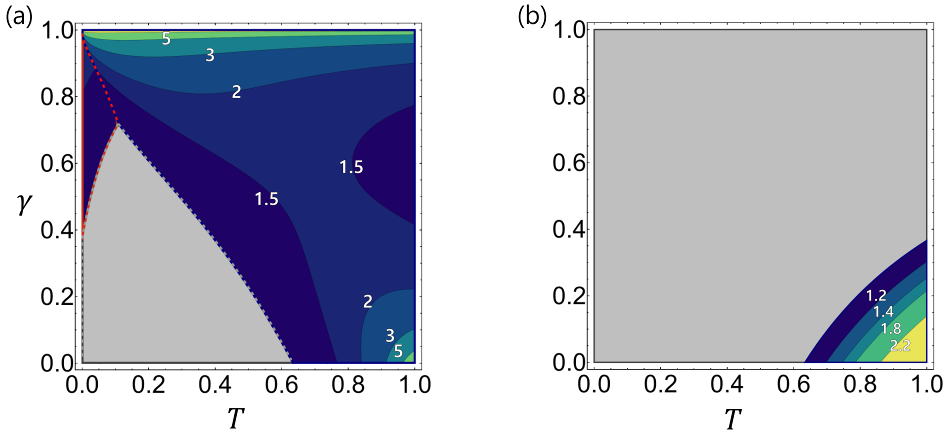

Fig. S1: Quantum enhancements achieved by the TMSV state as quantified by R_κ in (a) the original quantum setup and (b) the new quantum setup, for $n_{\text{th}} = 0$.

IV. PLOTS OF $\Delta\hat{T}$

This section provides plots of the standard deviations of the estimator, i.e. $\Delta\hat{T}$, achieved by the quantum and classical schemes. Figures S2 and S3 are plots of $\Delta\hat{T}$ in the original and alternative setups, respectively. Note that the results are for a single copy of the input state. For N copies, the standard deviation is divided by $\sqrt{N}$.

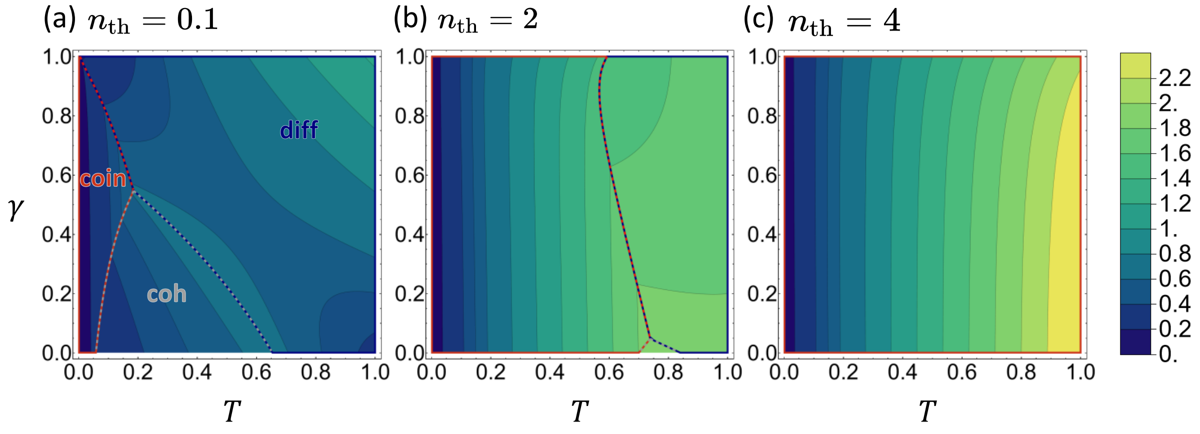

Fig. S2: $\Delta\hat{T}$ for the quantum and classical schemes in the original setup. The regions are divided according to their regions of dominance as in the main text.

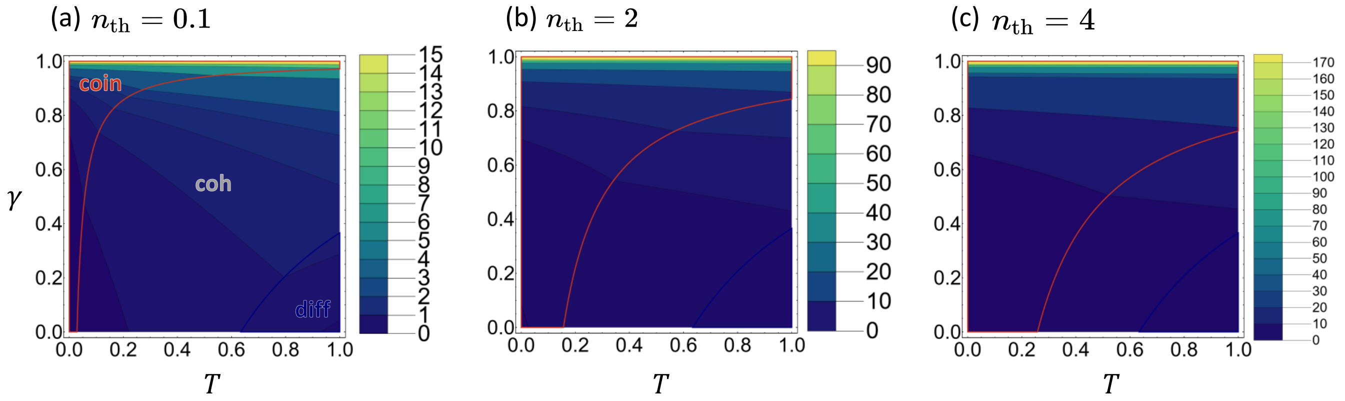

Fig. S3: $\Delta\hat{T}$ for the quantum and classical schemes in the alternative setup. The regions are divided according to their regions of dominance as in the main text.

[1] Braunstein, S. L. & Caves, C. M. Statistical distance and the geometry of quantum states. *Phys. Rev. Lett.* **72**, 3439 (1994).

[2] Banchi, L. Braunstein, S. L. & Pirandola, S. Quantum fidelity for arbitrary Gaussian states. *Phys. Rev. Lett.* **115**, 260501 (2015).

[3] Serafini, A. *Quantum Continuous Variables.* (CRC Press, 2017).

[4] Marian, P. & Marian, T. A. Uhlmann fidelity between two-mode Gaussian states. *Phys. Rev. A* **84**, 022340 (2012).

[5] Marian, P. & Marian, T. A. Quantum Fisher information on two manifolds of two-mode Gaussian states. *Phys. Rev. A* **93**, 052330 (2016).
